# Fiber Type Conversion by PGC-1α Activates Lysosomal and Autophagosomal Biogenesis in Both Unaffected and Pompe Skeletal Muscle

**DOI:** 10.1371/journal.pone.0015239

**Published:** 2010-12-13

**Authors:** Shoichi Takikita, Cynthia Schreiner, Rebecca Baum, Tao Xie, Evelyn Ralston, Paul H. Plotz, Nina Raben

**Affiliations:** 1 Arthritis and Rheumatism Branch, National Institute of Arthritis and Musculoskeletal and Skin Diseases, National Institutes of Health, Bethesda, Maryland, United States of America; 2 Light Imaging Section, Office of Science and Technology, National Institute of Arthritis and Musculoskeletal and Skin Diseases, National Institutes of Health, Bethesda, Maryland, United States of America; Fundação Oswaldo Cruz, Brazil

## Abstract

PGC-1α is a transcriptional co-activator that plays a central role in the regulation of energy metabolism. Our interest in this protein was driven by its ability to promote muscle remodeling. Conversion from fast glycolytic to slow oxidative fibers seemed a promising therapeutic approach in Pompe disease, a severe myopathy caused by deficiency of the lysosomal enzyme acid alpha-glucosidase (GAA) which is responsible for the degradation of glycogen. The recently approved enzyme replacement therapy (ERT) has only a partial effect in skeletal muscle. In our Pompe mouse model (KO), the poor muscle response is seen in fast but not in slow muscle and is associated with massive accumulation of autophagic debris and ineffective autophagy. In an attempt to turn the therapy-resistant fibers into fibers amenable to therapy, we made transgenic KO mice expressing PGC-1α in muscle (tgKO). The successful switch from fast to slow fibers prevented the formation of autophagic buildup in the converted fibers, but PGC-1α failed to improve the clearance of glycogen by ERT. This outcome is likely explained by an unexpected dramatic increase in muscle glycogen load to levels much closer to those observed in patients, in particular infants, with the disease. We have also found a remarkable rise in the number of lysosomes and autophagosomes in the tgKO compared to the KO. These data point to the role of PGC-1α in muscle glucose metabolism and its possible role as a master regulator for organelle biogenesis - not only for mitochondria but also for lysosomes and autophagosomes. These findings may have implications for therapy of lysosomal diseases and other disorders with altered autophagy.

## Introduction

Pompe disease (glycogen storage disease type II) is a rare genetic disorder that affects individuals at any age [1]. It is caused by deficiency of the enzyme acid alpha-glucosidase (GAA), which is essential for the degradation of glycogen to glucose in the acidic environment of the lysosomes. When GAA activity is absent or low, glycogen becomes trapped in the lysosomes in multiple tissues, but skeletal and cardiac muscles are the most vulnerable. The disease manifests with a broad clinical spectrum ranging from the severe rapidly progressive infantile form to milder late-onset variants [1]–[3]. The disease in infants, who have little or no enzyme activity, is characterized by profound hypotonia, feeding difficulties, and cardiomyopathy leading to death from cardiac failure within the first year of life [2], [4]. In the late-onset forms, caused by a partial enzyme deficiency, cardiac muscle is spared, but slowly progressive skeletal muscle weakness leads to wheelchair and ventilator dependence, and premature death from respiratory insufficiency [3].

A commercial drug, recombinant human GAA (rhGAA, Myozyme®; alglucosidase alpha, Genzyme Corporation, Framingham, MA), has recently become available for Pompe patients. The therapy, designed to replace the missing enzyme (enzyme replacement therapy; ERT), has profoundly changed the natural course of the disease in infants because of the impressive decrease in cardiac size and improvement in function. The patients survive significantly longer, but many still suffer from the persistent skeletal muscle myopathy and require assisted ventilation [5], [6]. In late-onset patients the therapy is claimed to stabilize the progression of the disease and improve the quality of life [7], [8], but incomplete clearance of the accumulated glycogen in skeletal muscle remains a concern in this form of the disease as well.

In our mouse model of the disease (KO), the poor skeletal muscle response to therapy is linked to a defect in the autophagic process. Macroautophagy (referred to as autophagy) is a major intracellular, lysosome-dependent, degradative pathway that involves the formation of autophagosomes which deliver cytoplasmic contents to lysosomes for degradation [reviewed in [9], [10]]. In both late-onset Pompe patients and KO mice, skeletal muscle fibers contain large areas of undegraded autophagic material [11]–[13]. In the KO, large pools of autophagic material are seen only in glycolytic type II muscle fibers, but not in oxidative type I fibers, which respond very well to therapy. Furthermore, in infants on ERT, a high proportion of type I fibers appears to be a good prognostic factor [14]. Therefore, a fiber type conversion by expression of PGC1-α seemed a reasonable therapeutic approach.

PGC-1α, which has recently emerged as a target of multiple physiological stimuli, is a member of the family of transcriptional cofactors of the nuclear receptor PPAR-γ (peroxisome proliferator-activated receptor γ) with a common function in the regulation of cellular energy metabolism. Multiple studies have shown that the PGC-1 family of co-activators, particularly PGC-1α, powerfully stimulates a variety of transcription factors and promotes the expression of genes involved in mitochondrial biogenesis and oxidative metabolism. Changes in PGC-1α level have been implicated in the pathogenesis of obesity, diabetes, neurological disorders, and cardiomyopathy as well as in ageing (reviewed in [15]–[17]).

Our interest in this molecule is related to its ability to convert fast (type II) glycolytic fibers to slow (type I) oxidative fibers which have increased oxidative capacity and mitochondrial mass [18]. We hypothesized that the fiber type conversion would make therapy-resistant type II fibers more amenable to therapy. In addition, PGC-1α has been shown to slow protein degradation in skeletal muscle [19] and to protect muscle from atrophy caused by ageing [20] or induced by denervation or fasting [21]. This anti-atrophic function of PGC-1α could possibly provide an additional benefit for Pompe disease, in which profound muscle wasting develops as the disease progresses.

We have generated a transgenic Pompe mouse model overexpressing PGC-1α in skeletal muscle (tgKO). Similar to what was reported in the wild type (WT) mice [18], an efficient fiber type conversion occurred in Pompe skeletal muscle. The autophagic buildup, a hallmark of Pompe disease in fast-twitch type II muscle, was no longer seen in the converted fibers, but unexpectedly, this genetic manipulation did not provide any additional therapeutic benefit. Analysis of PGC-1α transgenic Pompe mice, however, gave new insights into the pathogenesis of Pompe disease and into the role of PGC-1α in autophagosomal and lysosomal biogenesis.

## Results

We have previously shown that the autophagic buildup in muscles from KO mice is caused by a combination of increased production of autophagosomes and their inefficient resolution by lysosomes, leading to the accumulation of autophagic substrates - ubiquitinated (Ub)-proteins [22]. This buildup was found exclusively in glycolytic type II muscle fibers (also referred to as fast fibers), and is linked with resistance to ERT. The major objective of this study was to rid muscle cells of this autophagic buildup by converting type II fibers into therapy-responsive type I fibers which have no autophagic accumulation.

### Expression of PGC-1α in skeletal muscle results in a shift from fast to slow muscle profile, but not a complete fiber type conversion

**Figure 1:**
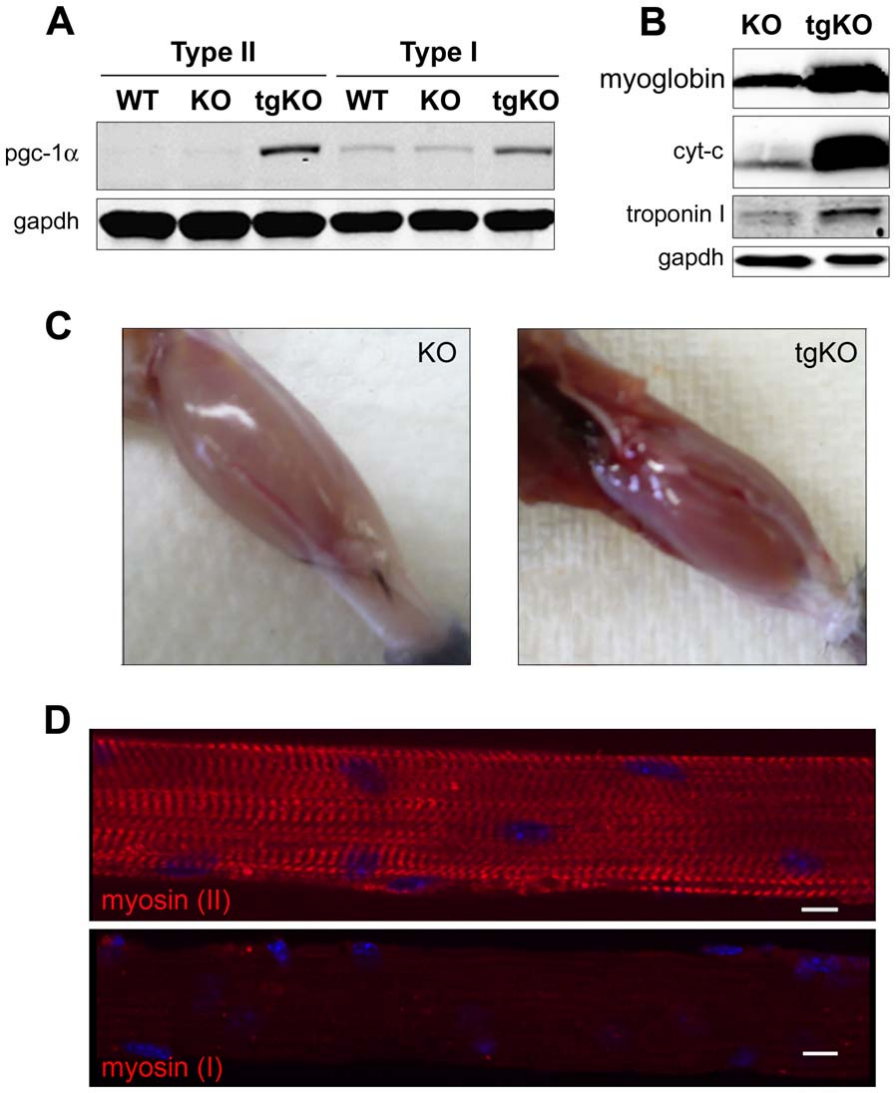
Characterization of muscle-specific tgKO mice. (**a**) Western blotting of protein lysates from white gastrocnemius (type II) and soleus muscle (type I) derived from WT, KO, and tgKO mice with PGC-1α antibody. The transgene is expressed in both type I and type II fibers. Muscle samples were taken from 3–6 month-old mice. Two WT, five KO and five tgKO mice were analyzed. (**b**) Western blot analyses of different proteins in gastrocnemius muscle of KO and tgKO mice. GAPDH was used as loading control. (**c**) Appearance of gastrocnemius muscle fibers in KO and tgKO mice. (**d**) Single fibers were stained for the fast (myosin II) and slow (myosin I) isoforms of myosin heavy chain. The fibers were isolated from psoas muscle (which normally contains mostly type II fibers) of a tgKO mouse. Bar: 10 µm.

The expression of endogenous PGC-1α in fast (white gastrocnemius) and slow (soleus) muscles from KO mice was similar to that in the WT mice; in both strains higher levels of PGC-1α were found in slow muscles (**Figure 1A**). In tgKO mice the transgene was highly expressed in both fast and slow muscles, with the increase most pronounced in fast muscles. As expected, transgenic expression of PGC-1α in skeletal muscle of Pompe mice resulted in a fiber type transition from glycolytic to oxidative fibers; white gastrocnemius muscles, normally rich in type II fibers, showed a red color due to a high concentration of myoglobin, an elevated level of cytochrome *c*, and an increased production of specialized type I fiber contractile protein troponin I (slow) - all characteristic of type I fibers (**Figures 1B and 1C**). Thus, the hallmarks for muscle fiber type switching observed in PGC-1α transgenic mice [18] are also seen in PGC-1α transgenic mice on a KO background. The PGC-1α converted fibers, however, still stained for the fast isoform of myosin heavy chain (myosin II, expressed in fast-twitch fibers) and not for the slow isoform (**Figure 1D**), suggesting that other factors play a role in fiber type identity. It has been previously shown that PGC-1α is not the only determinant of the fiber type: a significant number of type I and IIa fibers still remained in muscle-specific PGC-1α - knockout mice [23]. The incomplete switch in fiber type transition from glycolytic to oxidative fibers in our experiments is also supported by only partial changes in the positioning of the Golgi complex and microtubules in the converted fibers (Supplementary Material, **Figures S1 and S2**).

### Expression of PGC-1α in skeletal muscle results in the up-regulation of autophagy

**Figure 2:**
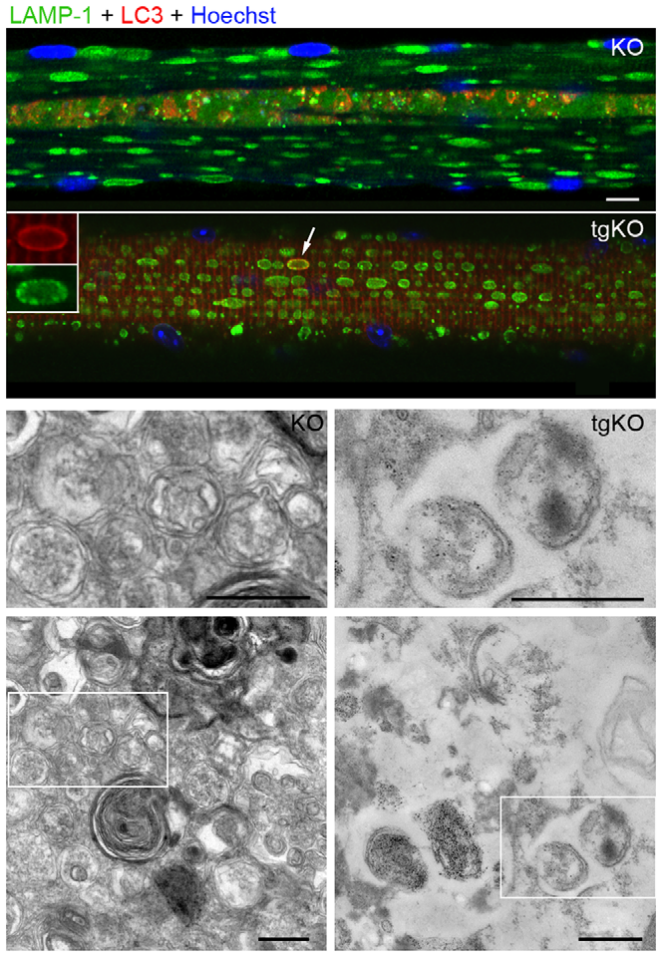
The absence of the autophagic buildup in converted fibers of tgKO. Immunostaining of single psoas (fast) fibers for LAMP-1 and LC3. A centrally located region with multiple LC3-positive structures, which represents autophagic buildup, is found in the KO, but not in the tgKO fiber. An isolated LC3-positive autophagosome (arrowhead and inset) can be found in the converted fiber from the tgKO. Electron microscopy (B&W panels) shows autophagosomes in psoas fibers in both KO and tgKO mouse lines. Magnification of the areas marked by the white boxes is shown in the upper panels. Muscle samples were taken from 5 month-old mice. Bar: 10 µm (immunofluorescence) and 500 nm (EM).

As in type I fibers of the KO, no autophagic buildup was observed in gastrocnemius or psoas muscles from tgKO (**Figure 2**). Another similarity between the converted tgKO fibers and type I KO fibers was the presence of occasional autophagosomes (**Figure 2**) or pockets of autophagosomes (Supplementary Material, **Figure S3**) as evidenced by immunostaining with a specific autophagosomal marker, microtubule-associated light chain protein 3 (LC3).

**Figure 3:**
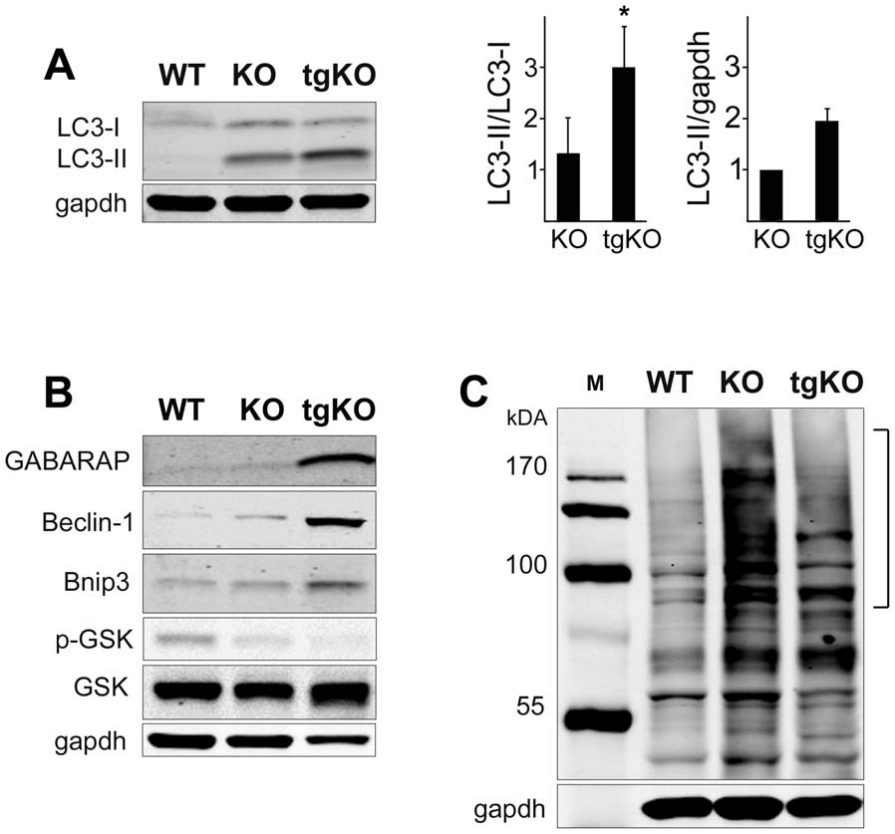
Induction of autophagy in converted fibers of the tgKO mice. (**a**). Western blotting of protein lysates from gastrocnemius (fast) muscles from WT, KO, and tgKO mice with LC3 antibody. The effect of the transgene on autophagy was assessed as an increase in the LC3-II/LC3-I ratio (left graph; * p<0.0011), and as a fold increase of LC3-II/gapdh ratio (right graph) in the tgKO compared to the KO. Five mice were analyzed in each group. (**b**)**.** Western blotting of protein lysates from gastrocnemius (fast) muscles from WT, KO, and tgKO mice with the indicated antibodies. GABARAP, like LC3-II, has been shown to localize to the autophagosomal membrane [55]; Beclin-1 is part of the Class III PI3K complex which participates in autophagosome formation [56]; Bnip3, a hypoxia-inducible member of the Bcl-2 protein family, is thought to induce autophagy by releasing Beclin-1 from the BCL-2 complex [57]. A markedly decreased phosphorylation of GSK-3β (on Ser^9^) is observed in muscle from KO and tgKO mice without a decrease in the amount of total GSK-3β. Decreased phosphorylation of GSK-3β leads to activation of the kinase, which is a positive regulator of autophagy [24]. (**c**). Western blotting of protein lysates from gastrocnemius (fast) muscle with anti-ubiquitin (FK2) antibody. The amount of high molecular weight ubiquitinated proteins (marked by a bracket) is much lower in the converted fibers of tgKO compared to the KO. M: protein marker. Data shown are representative of at least four independent experiments.

Counterintuitively, the absence of autophagic buildup was associated with induction rather than suppression of autophagy in tgKO when compared to KO. A robust increase in the autophagosomal membrane-bound form of LC3 (LC3-II) and in several other autophagy-related proteins such as GABARAP (gamma-aminobutyric acid receptor-associated protein), Beclin-1, and BNIP3 (Bcl-2/E1B 19 kDa interacting protein) all point to an up-regulation of autophagy (**Figures 3A and 3B**). In addition, a recently recognized inducer of autophagy, glycogen synthase kinase (GSK) [24], was activated (less phosphorylated) in PGC-1α converted muscles when compared to the KO (**Figure 3B**). The induction of autophagy without the formation of autophagic buildup indicates more efficient resolution of autophagosomes by lysosomes in tgKO when compared to the KO; a significant decrease in the amount of Ub-proteins attests to this view (**Figure 3C**).

**Figure 4:**
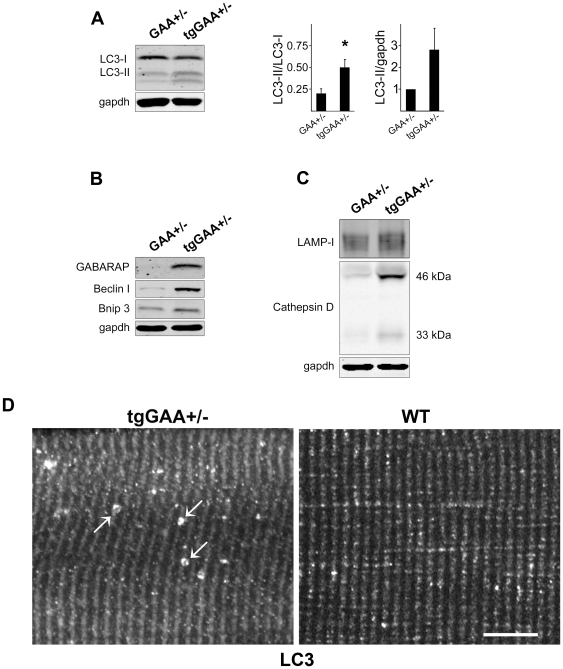
Induction of autophagosomal and lysosomal biogenesis in control PGC-1α transgenic mice. (**a**)**.** Western blotting of protein lysates from gastrocnemius (fast) muscles from control GAA+/− and tgGAA+/− mice with LC3 antibody. The effect of the transgene on autophagy was assessed as an increase in the LC3-II/LC3-I ratio (left graph; * p<0.005), and as a fold increase of LC3-II/gapdh ratio (right graph) in the transgenic compared to non-transgenic controls. Four mice were analyzed in each group. Note the batch of LC3 antibodies used for these experiments resulted in the appearance of an additional lower band. (**b**) Western blotting of protein lysates from gastrocnemius (fast) muscles from control GAA+/− and tgGAA+/− mice with the indicated antibodies showing increased levels of autophagy-related proteins – GABARAP, Beclin-1, and Bnip3. (**c**). Western blotting of protein lysates from gastrocnemius (fast) muscles from control GAA+/− and tgGAA+/− mice with LAMP-1 and cathepsin D antibodies showing increased levels of two lysosomal proteins. Note that cathepsin D runs as two bands of 46 and 33 kDa. Four mice were analyzed in each group. (**d**) Single fibers (type II; psoas) from tgGAA+/− and WT mice stained for LC3 (the image is shown in black and white). LC3-positive vesicular structures (arrows) represent autophagosomes. Structures like these are never seen in WT or GAA+/− muscles. Bar: 10 µm.

To extend these observations to normal skeletal muscle we used PGC-1α transgenic mice on the GAA+/− background (tgGAA+/−) as controls; GAA+/− mice are phenotypically, morphologically, and biochemically normal. In these control mice autophagy was also induced as shown by an increase in LC3-II, Beclin-1, and BNIP3 when compared to the GAA+/− (**Figures 4A and 4B**). Furthermore, LC3-positive autophagosomes, vesicular structures not observed in WT or GAA+/− muscle, can be found in tgGAA+/− muscle (**Figure 4D**).

### Expression of PGC-1α in skeletal muscle results in increased lysosomal number and glycogen load

**Figure 5:**
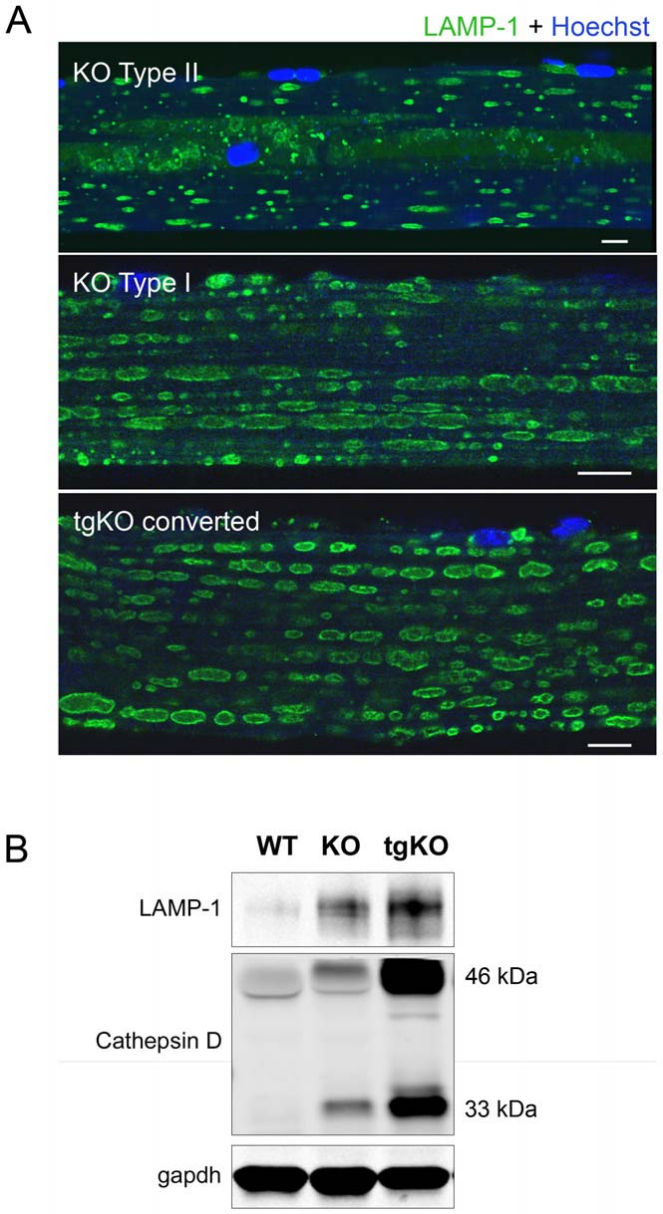
Induction of lysosomal biogenesis in converted fibers from the tgKO. (**a**) Single muscle fibers were stained for LAMP-1. The position of lysosomes in the converted type II fibers from tgKO is similar to those in type I fibers. Bar: 10 µm. (**b**) Western blotting of protein lysates from gastrocnemius (fast) muscles from WT, KO, and tgKO mice with LAMP-1 and cathepsin D antibodies showing increased levels of two lysosomal proteins. Two WT, six KO and six tgKO mice were analyzed.

The number of lysosomes appeared to dramatically increase in gastrocnemius and psoas muscles from tgKO when compared to the KO, and the distribution of the lysosomes was reminiscent of that in type I soleus muscle (**Figure 5A**). Consistent with the increase in the number of lysosomes in the tgKO, levels of the lysosomal associated membrane protein 1 (LAMP-1) and the lysosomal enzyme cathepsin D were elevated (**Figure 5B**). An increase in these lysosomal markers was also observed in control tgGAA+/− mice (**Figure 4C**). Thus, PGC-1α not only stimulated mitochondrial but also autophagosomal and lysosomal biogenesis in both normal and diseased muscle.

**Figure 6:**
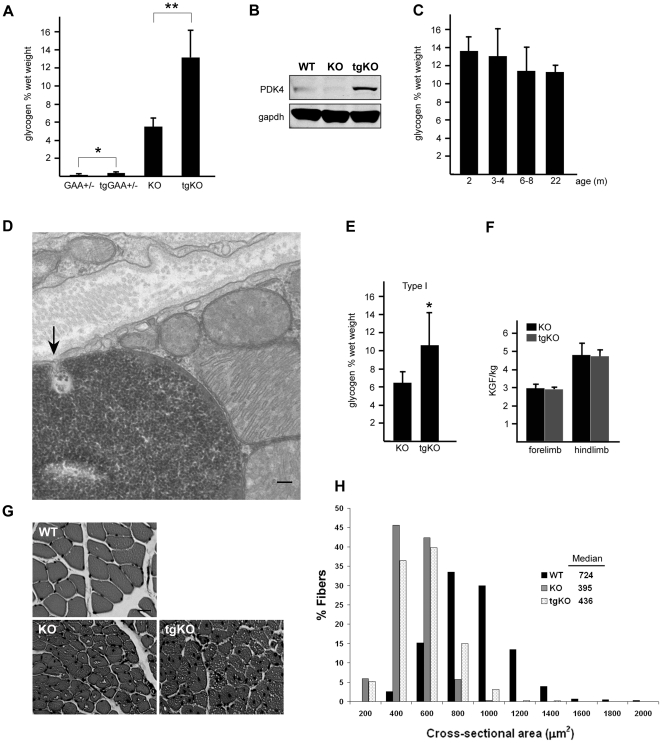
Effect of PGC-1α on muscle glycogen levels and muscle strength. (**a**). Gastrocnemius (fast) muscles were used for glycogen measurement. Note a striking increase in the amount of glycogen in muscle from tgKO compared to KO; * p<0.03; ** p<0.001. (**b**). Western blotting of protein lysates from gastrocnemius (fast) muscles from WT, KO and tgKO mice with PDK4 antibody. (**c**). Glycogen levels in gastrocnemius (fast) muscles from tgKO mice at different ages (n = 5 for 2 m; n = 11 for 3–4 m; n = 6 for 6–8 m; and n = 3 for 22 m). (**d**). Electron microscopy of soleus muscle from a tgKO mouse showing glycogen particles (arrow) entering a single membrane lysosome which is already filled with glycogen. Bar: 100 nm. (**e**). An increase in the amount of glycogen is also observed in soleus (type I slow) muscle of the tgKO mice; * p<0.04. (**f**). Muscle strength of KO and tgKO mice. Each value is the mean ± sd. from 5 animals. Five consecutive fore- and hindlimb strength measurements were recorded daily for each mouse over a 5-day-period. The maximum values for each day were used for the analysis. The data were normalized to body weight and expressed as KGF/kg. Five month-old male mice were used for the experiments. (**g**) H & E-stained sections of tibialis anterior muscle from 1.5 year-old WT, KO, and tgKO. Both KO and tgKO fibers are atrophic compared to the WT. Bar: 20 µm. (**h**) Histogram showing the distribution of cross-sectional areas of WT, KO, and tgKO muscle fibers.

The increased number of lysosomes in gastrocnemius muscle of tgKO mice was accompanied by a rise in glycogen load to a strikingly high level when compared to the KO (from 5.6±0.9 to 13.7±3.1% wet weight; **Figure 6A**), suggesting a role of PGC-1α in glycogen metabolism in muscle. Indeed, we found a modest increase in cytoplasmic glycogen in control tgGAA+/− mice (0.099±0.063 and 0.216±0.086% wet weight in GAA+/− and tgGAA+/− respectively; p<0.03; **Figure 6B**), and an increase in the level of PDK4, an inhibitor of muscle glucose oxidation, in tgKO compared to KO or WT (**Figure 6B**).

Thus, a small additional load of cytoplasmic glycogen turned into a dramatic increase in the amount of glycogen trapped in the lysosomes in the tgKO, in which GAA is absent. This large amount of lysosomal glycogen in tgKO suggests that the transit of glycogen through lysosomes is a very active process, which cannot be appreciated when GAA functions normally. Glycogen in the tgKO reaches its maximum level by 2 months of age (the earliest time studied; **Figure 6C**). These high levels are likely due to enhanced autophagy, a presumed pathway for glycogen delivery to the lysosomes. Electron microscopy provided a snapshot of yet another route of glycogen trafficking to the lysosomes: direct invagination of the lysosomal membrane, a process known as microautophagy (**Figure 6D**).

### Expression of PGC-1α in skeletal muscle does not affect muscle strength and fiber size

An increase in lysosomal glycogen was also seen in soleus muscle of tgKO (**Figure 6E**), but despite the higher level of glycogen in tgKO muscle, the overall strength of these mice was no different than that of the KO (**Figure 6F**). Similarly, the cross-sectional area of the converted fibers was only slightly increased in the tgKO compared to KO. Both tgKO and KO strains exhibited profound atrophy when compared to the WT (**Figures 6G and 6H**).

### Fiber type conversion with PGC-1α does not improve enzyme replacement therapy

**Figure 7:**
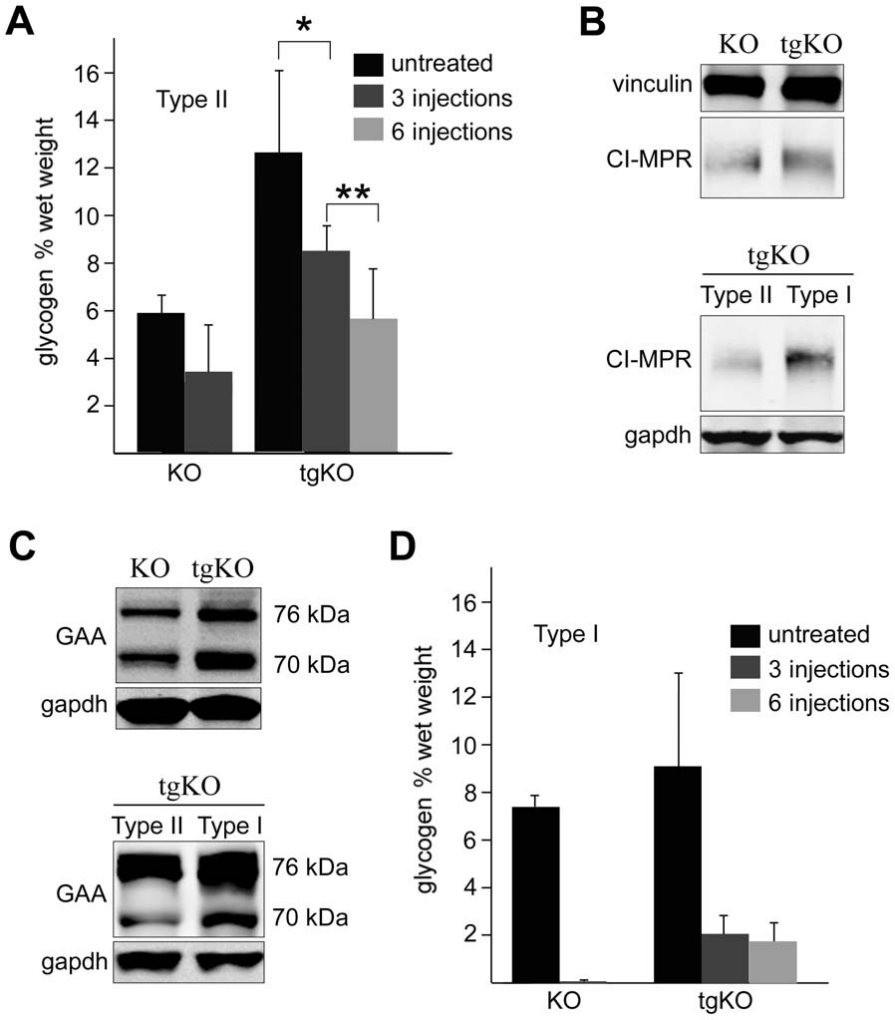
Effect of ERT on glycogen clearance in skeletal muscle of KO and tgKO mice. (**a**) Quantitation of glycogen levels in gastrocnemius (type II fast) muscle from KO and tgKO mice before and after ERT. Values are the mean ± sd.; n = 3 and n = 13 for the untreated KO and tgKO mice respectively; n = 2 and n = 7 for the experiments with 3 injections for the KO and tgKO mice respectively; 6 tgKO mice were used for 6 injections of the rhGAA; * p<0.007; ** p<0.011. (**b** and **c**). Western blotting of protein lysates from gastrocnemius (fast) muscle of KO and tgKO mice and from soleus (slow) muscle of tgKO with CI-MPR and GAA antibody. The density of the CI-MPR in fast muscle of tgKO is similar to that in the KO (**Fig. 7b top**). The amount of processed rhGAA (76 and 70 kDa lysosomal forms) in fast muscle of tgKO is no less than in the KO (**Fig. 7c top;** in the sample shown the amount is even more). Similar to what was previously shown for the KO [26], the receptor density and the amount of processed rhGAA in tgKO are higher in slow than in fast muscle (**Figs. 7b,c bottom**). Two KO and four tgKO mice were analyzed in each group. (**d**). Quantitation of glycogen levels in soleus muscle from KO and tgKO mice before and after ERT. Values are the mean ± sd.; n = 3 and n = 6 for the untreated KO and tgKO mice respectively; n = 2 and n = 7 for the experiments with 3 injections for the KO and tgKO mice respectively; 6 tgKO mice were used for 6 injections of the rhGAA.

In our previous studies genetic suppression of autophagy in skeletal muscle of Pompe mice led to complete clearance of muscle glycogen after three injections of the recombinant enzyme (rhGAA) [25]. The same regimen in tgKO resulted in only a 40% reduction of glycogen in gastrocnemius muscle (**Figure 7A**). This outcome raised the question of whether PGC-1α negatively affected the uptake and processing of the administered recombinant enzyme. We have found no difference in the density of the cation-independent mannose-6-phosphate receptor (CI-MPR) (**Figure 7B**), which is responsible for the uptake and trafficking of rhGAA to the lysosomes, and in the amount of 76 and 70 kDa lysosomal forms (**Figure 7C**) in the tgKO and KO mice. Consistent with our previous data in the KO mice [26], the receptor density and the amount of processed enzyme in tgKO were higher in slow than in fast muscle (**Figures 7B and 7C**).

Since the basal level of glycogen in the tgKO is significantly higher than in the KO, we extended the therapy by three additional injections and observed a further drop in glycogen, but a significant amount of glycogen remained (**Figure 7A**). Furthermore, ERT, which clears glycogen very efficiently in KO soleus muscle, failed to normalize glycogen even in this muscle in the tgKO (**Figure 7D**). Thus, the absence of the autophagic buildup does not overcome the negative effect of the excess of glycogen in both gastrocnemius and soleus muscles in tgKO. Although the results were disappointing, it is important to note that the amount of glycogen cleared from tgKO muscle exceeded the amount that typically accumulates in muscle of the KO (**Figure 7A**).

**Figure 8:**
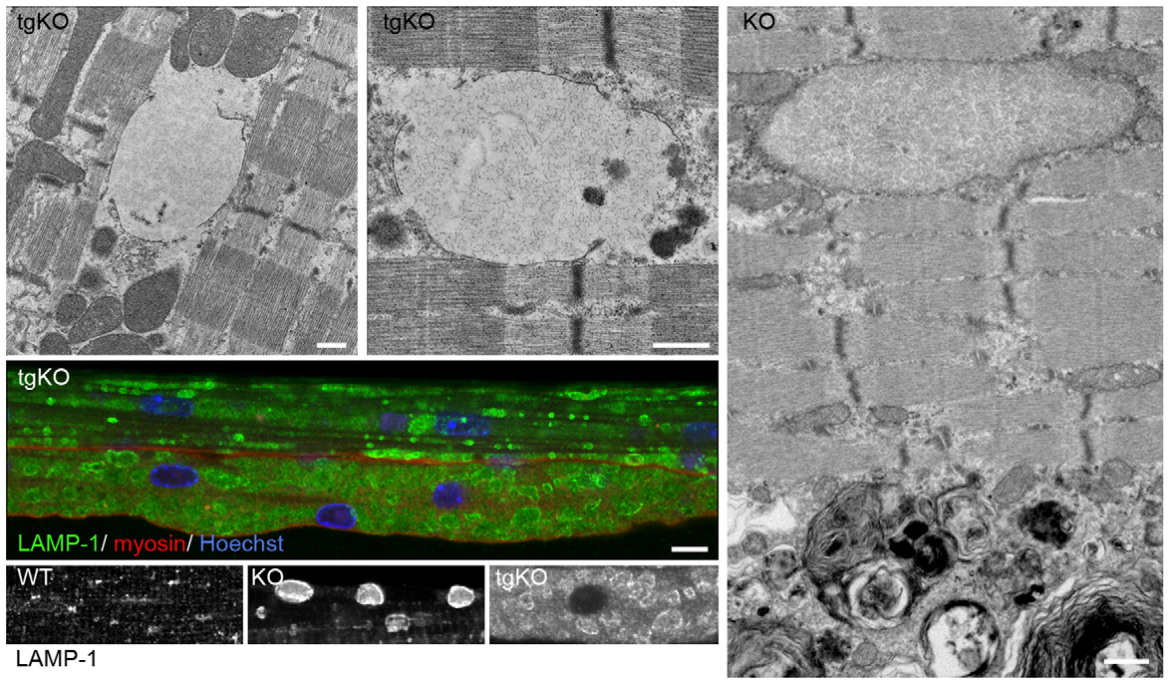
Lysosomal rupture in the converted fibers of tgKO. Electron microscopy of gastrocnemius fast muscles of 5 month-old tgKO (two top panels) and KO (right panel) mice. Several break points of the lysosomal membrane can be seen in the tgKO. Lysosomal membrane is preserved in the KO; autophagic buildup can be clearly seen. Bar: 500 nm. Middle panel: Single fibers (psoas muscle) from tgKO stained with LAMP-1 (green) and myosin-slow (red) showing multiple lysosomes with an irregular shape and broken borders in the lower fiber. Nuclei are stained with DAPI. Bar: 20 µm. Bottom panels: Single fibers (psoas muscle) from WT, KO, and tgKO stained with LAMP-1 (shown in white). Lysosomes appear as dot-like structures in the WT and as well-defined vesicles in the KO; only remnants of lysosomal membranes are detected in the tgKO.

We considered the possibility that excess glycogen in tgKO might lead to lysosomal rupture, a phenomenon observed in infantile patients with a glycogen burden much closer to that in tgKO than in the KO. Electron microscopy of skeletal muscle in tgKO indeed showed that not all glycogen was confined within the membrane-bound lysosomes (**Figure 8**; an additional image is provided in Supplementary Material, **Figure S4**). Multiple broken lysosomes which release glycogen into the cytoplasm were observed by EM. This finding is supported by immunostaining of single muscle fibers for LAMP-1 which shows remnants of lysosomal membranes (**Figure 8**).

## Discussion

The experiments described in this paper were motivated by the need to improve the efficacy of enzyme replacement therapy in a metabolic myopathy, Pompe disease. Multiple factors contribute to the difficulties in treating skeletal muscle: the sheer mass of muscle tissue; the low density of the receptor responsible for the uptake and delivery of the recombinant enzyme to the lysosomes; and the diversion of the enzyme to the liver. We have previously reported that dysfunctional autophagy and accumulation of autophagic debris in fast muscles of the Pompe model add considerably to these difficulties [27]. A profound abnormality in the autophagic pathway also occurs in skeletal muscle in humans with the disease [13], [28]. In late-onset patients, as in the mouse model, the enormous autophagic buildup causes greater skeletal muscle damage than the enlarged lysosomes outside the autophagic regions. Therefore, we looked for ways to eliminate the autophagic buildup in the hope of improving the effect of therapy. Indeed, our previous data showed that genetic suppression of autophagy combined with ERT resulted in complete removal of muscle glycogen [25].

Converting fast to slow fibers, which in the KO do not have autophagic buildup and respond well to therapy, looked like an attractive and more physiological approach. This approach would avoid the need for the genetic suppression of autophagy in skeletal muscle, a condition that has been found to be associated with some abnormalities [29]–[31]. Both developing and adult skeletal muscle have considerable plasticity with respect to fiber-type switching [32]. For example, endurance training stimulates mitochondrial biogenesis and a switch from fast to slow fibers. We have attempted the fiber type conversion in Pompe muscles by transgenic expression of PGC-1α, a factor which drives the slow muscle metabolic program [18].

As expected, transgenic PGC-1α KO mice, like transgenic PGC-1α WT mice [18], showed conversion of glycolytic fibers into mitochondria-rich oxidative fibers. The converted fibers were atrophic with the degree of atrophy similar to that seen in the KO muscle. In the literature, the data on the effect of PGC-1α on muscle fiber size do not paint a clear picture. Overexpression of PGC-1α was shown to inhibit denervation atrophy and protect skeletal muscle against atrophy induced by expression of FOXO3 [21]. An anti-atrophic effect of PGC-1α in skeletal muscle was also observed during ageing [20]. On the other hand, Miura et al. [33] reported that overexpression of PGC-1α in skeletal muscle resulted in a marked depletion of ATP leading to atrophy, especially in type IIB-rich muscles. By contrast, we found a negligible effect of PGC-1α on muscle atrophy in the tgKO muscle.

The fiber type conversion resulted in a striking disappearance of the autophagic buildup. How and why the autophagic buildup is formed in type II fibers of the KO is puzzling. The buildup is very particular in its content and position; it is commonly located in the core of the fibers, and it contains a subset of clustered lysosomes with compromised membranes that appear different from those in the rest of the fiber. It was not clear whether it is the intrinsic properties of this subset of lysosomes that make them incapable of resolving the incoming autophagosomes or whether it is the metabolic/contractile properties of the fiber itself which create this unusual pathology. Since the conversion of fast muscle into muscle with slow profile in the KO mice completely eliminated the autophagic inclusions, the latter scenario appears much more plausible.

Despite the absence of autophagic buildup, the therapy was not successful in tgKO, most likely because of the unexpectedly high glycogen burden leading to lysosomal rupture and release of glycogen into the cytoplasm where its fate and possible effects on muscle function remain to be determined. The reported data on the role of PGC-1α in muscle glycogen and glucose metabolism is somewhat controversial. In transgenic mice, PGC-1α was shown to suppress glucose transport [34], and to decrease insulin-stimulated muscle glucose uptake in mice on a high-fat diet [35]. In contrast, expression of PGC-1α resulted in an induction of glucose transport in muscle cells *in vitro* [36] and *in vivo* leading to an increase in cytoplasmic glycogen [37]. This increase in muscle glycogen stores in skeletal muscle of PGC-1α transgenic mice was also due to the inhibition of glycolysis and down-regulation of glycogen phosphorylase, the enzyme responsible for the degradation of glycogen in the cytoplasm [37].

We, too, found an increase in glycogen in control PGC-1α transgenic mice. Although this increase is statistically significant compared to the controls, the absolute levels of the accumulated cytoplasmic glycogen are still barely above the detectable threshold. This slight increase, however, leads to an early, massive accumulation of lysosomal glycogen when the transgene is placed on the GAA KO background suggesting that a significant portion of cytoplasmic glycogen rapidly ends up in the lysosomes, where it cannot be digested. The muscle pathology in tgKO is comparable to that in skeletal muscle of infantile Pompe patients who have much greater glycogen load than the knockout mice [38].

Glycogen is thought to reach the lysosomes, at least in part, via the autophagic pathway. Glycogen autophagy and lysosomal degradation of glycogen to glucose have been shown in the liver [39], [40] and in skeletal muscle [41], [42] during the early postnatal period as a response to a high demand for this sugar. We have previously demonstrated that genetic suppression of autophagy in skeletal muscle significantly reduced the glycogen burden in KO mice [25]. Consistent with these data, we now show that the increase in lysosomal glycogen load in tgKO is associated with an up-regulation of autophagy even greater than that seen in KO muscles.

There are only a limited number of reports on the role of PGC-1α in the regulation of autophagy. A positive correlation between an increase in PGC-1α and autophagy has been shown in lipopolysaccharide-treated neonatal rat cardiomyocytes [43]. Similarly, activation of PPAR-γ induced autophagy in breast cancer cells through upregulation of the HIF-1α protein and BNIP3 [44]. On the other hand, PGC-1α was shown to inhibit autophagic/lysosomal protein degradation in myotubes [19] and to suppress autophagy in muscles in aged PGC-1α transgenic mice [20]. In our system PGC-1α clearly induced autophagy in both control and KO mice.

Another finding related to the function of PGC-1α itself is a significant increase in the number of lysosomes which became obvious because of the KO background. Thus, our data suggest that PGC-1α is a regulator not only of mitochondrial but also of lysosomal and autophagosomal biogenesis. A combination of enhanced lysosomal capacity and increased production of autophagosomes without autophagic buildup resulted in more efficient disposal of autophagic cargo, in particular Ub-proteins, in tgKO than in KO mice.

Clearance of potentially toxic Ub-substrates is a major problem in neurodegenerative diseases, and the induction of autophagy has emerged as a therapeutic approach designed to rid the cells of these abnormal protein aggregates [reviewed in [45]]. Our data on the upregulation of autophagy by PGC-1α suggest that pharmacological activation of this molecule might have a therapeutic benefit for a range of neurodegenerative diseases caused by the accumulation of such aggregates.

Finally, the failure of ERT in tgKO mice may be due to very high, non-physiologic levels of PGC-1α transgene expression, which even exceed the endogenous levels of PGC-1α in type I soleus muscle (Fig. 1). (The levels of PGC-1α expression in tgKO mice are higher compared to those in the “original” PGC-1α strain [18], which was used for crosses to the KO; these high levels could be due to the differences in the background). It has been shown that modest PGC-1α expression in skeletal muscle increased insulin sensitivity [46] whereas excessive PGC-1α expression in transgenic mice rendered skeletal muscle resistant to insulin [35]. A moderate increase in PGC-1α can be achieved by exercise (which can influence fiber type distribution) as demonstrated in humans [17] and in rats [47]. Other routes of fiber type conversion may be more successful. It has recently been shown that the expression of the myosin intronic microRNA (miR-499) in skeletal muscle powerfully induced the conversion from fast to a slower myofiber type [48]. Thus, the disappointing outcome of therapy in tgKO cannot be viewed as a final verdict on the merits of fiber type conversion.

## Materials and Methods

### Generation and genotyping of transgenic GAA-/- mice expressing PGC-1α in skeletal muscle (tgKO)

Transgenic MCK- PGC-1α mice, generously provided by Dr. Bruce M. Spiegelman (Dana-Farber Cancer Institute and the Department of Cell Biology, Harvard Medical School, Boston, MA), express PGC-1α in skeletal muscle at physiological levels under the control of the muscle creatine kinase (MCK) promoter [18] which is particularly active in type II fibers. These mice were crossed with Pompe knockout mice (KO) [49] to produce Pompe mice expressing PGC-1α in skeletal muscle (referred to as tgKO). The colony contained ∼120 mice, which were maintained on a standard diet. The clinical signs of muscle disease were monitored in ∼50 mice. Genomic DNA was isolated from tail clips using the iPrep™ ChargeSwitch® gDNA tissue Kit (Invitrogen, Carlsbad, CA) or the QuickGene DNA tissue kit (FUJIFILM, Tokyo, Japan) according to the manufacturers' instructions. The presence of the transgene is indicated by a ∼330 bp PCR product obtained with the primer pair: 5′gcaggatcacataggcaggatgtggcc 3′ (MCK promoter)/5′ ggaagatctgggcaaagaggctggtcc 3′ (PGC-1α). The *GAA +/−*, and *GAA−/−* alleles were detected as described [49]. PGC-1α transgenic mice on a GAA +/− background (tgGAA+/−) were used as controls. GAA+/− are phenotypically normal and do not accumulate any lysosomal glycogen.

### Enzyme replacement therapy

Two and a half month-old KO (n = 3) and tgKO (n = 7) mice received 3 intravenous injections of recombinant human α-glucosidase (rhGAA; Myozyme®, Genzyme Corporation, Framingham, MA; provided under a CRADA between the NIH and the Genzyme Corporation) at a dose of 100 mg/kg every other week. To diminish a hypersensitivity reaction, diphenhydramine hydrochloride was injected intraperitoneally at a dose of 5 mg/kg 15 minutes before the second and third injections of rhGAA as described [50]. The mice were sacrificed 5 days after the last injection. Six additional tgKO mice received 6 injections of rhGAA; these mice were sacrificed three days after the last injection.

### Isolation of fixed single muscle fibers and immunofluorescence microscopy

Muscle fixation, isolation of single fibers, and immunostaining are described [51]. Briefly, muscles were removed immediately after sacrifice and pinned to Sylgard-coated dishes for fixation with 2% paraformaldehyde in 0.1 M phosphate buffer for 1 h, followed by fixation in methanol (−20°C) for 6 min. Single fibers were obtained by manual teasing. Fibers were placed in a 24-well plate in M.O.M. Blocking Reagent (Vector Laboratories, Burlingame, CA) for 1 h. The fibers were then permeabilized, incubated with primary antibody overnight at 4°C, washed, incubated with secondary antibody for 2 h, washed again, and mounted in Vectashield (Vector Laboratories) on a glass slide. At least 3 animals from each genotype were used to obtain single muscle fibers for immunostaining. For each immunostaining and for confocal analysis, at least 20 fibers were isolated.

### Electron Microscopy

Muscles were fixed and treated as described [22] except that in some cases osmication was done in the presence of 1.5% potassium ferrocyanide ("reduced osmium") for darker glycogen staining [52].

### Western blot

Whole muscle tissues were homogenized in RIPA buffer [PBS containing 1% NP40, 0.5% sodium deoxycholate, 0.1% SDS and a protease inhibitor cocktail tablet (Roche Diagnostics, Indianapolis, IN)]. Samples were centrifuged for 30 min at 13,000 rpm at 4°C. Protein concentrations of the supernatants of the total lysates or soluble fractions were measured using the Bio-Rad Protein Assay (Bio-Rad Laboratories, Inc., Hercules, CA). Equal amounts of protein were run on SDS-PAGE gels (Invitrogen, Carlsbad, CA) followed by electro-transfer onto nitrocellulose membranes (Invitrogen, Carlsbad, CA). Membranes were blocked in 1∶1 PBS and Odyssey Blocking Buffer (LI-COR Biosciences, Lincoln, NE), incubated with primary antibodies overnight at 4°C, washed, incubated with secondary antibodies, and washed again. Blots were scanned on an infrared imager (LI-COR Biosciences).

### Antibodies

The following primary antibodies were used for Western blots and immunostaining of fixed fibers: rabbit antiserum to bovine cation independent mannose-6-phosphate receptor (CI-MPR; a gift from Dr. Stuart Kornfeld, Washington University School of Medicine, St. Louis, MO); rabbit polyclonal anti-hGAA (Genzyme Corp. Framingham, MA); rabbit anti-LC3B (microtubule-associated protein 1 light chain 3) (Sigma, St. Louis, MO); rat anti-mouse LAMP-1 (lysosomal-associated membrane protein 1) (BD Pharmingen, San Diego, CA); mouse anti-poly-ubiquitinated conjugates (FK2) (BIOMOL International, L.P., Philadelphia, PA); rabbit polyclonal anti-Beclin-1 (Cell Signaling Technology, Inc., Danvers, MA); rabbit monoclonal anti-GSK-3β, rabbit monoclonal anti-phospho-GSK-3β (Ser9) (Cell Signaling Technology); rabbit polyclonal anti-GABA_A_ receptor associated protein (Millipore, Billerica, MA); rabbit polyclonal anti-Bnip3 (Abcam, Cambridge, MA); rabbit polyclonal anti-PGC-1 (Millipore); goat polyclonal anti-cytochrome c (Santa Cruz Biotechnology, Inc., Santa Cruz, CA); rabbit polyclonal anti-myoglobin (Santa Cruz Biotechnology); rabbit polyclonal anti-troponin type I (Abcam); goat anti-mouse cathepsin D (R&D Systems, Inc., Minneapolis, MN); rabbit polyclonal anti-PDK4 (Abcam); mouse monoclonal anti-GM130 (BD Pharmingen); mouse monoclonal anti-alpha tubulin (Sigma); mouse monoclonal anti-myosin (skeletal, slow) and anti-skeletal myosin (fast) (this antibody does not distinguish between type II subtypes) (Sigma); mouse monoclonal anti-vinculin (Sigma) and mouse monoclonal anti-GAPDH antibody (Abcam) served as loading controls. Alexa Fluor-conjugated antibodies (Molecular Probes, Eugene, OR) were used as secondary antibodies.

### The source of fast and slow muscle fibers

Gastrocnemius and psoas muscles in mice are fast muscles. Psoas and the white part of gastrocnemius muscles are good sources of glycolytic fast-twitch type II fibers (also referred to as fast), whereas soleus muscle is a good source of oxidative slow-twitch type I fibers (also referred to as slow) [38].

### Glycogen measurement and light microscopy

Glycogen concentration in skeletal muscle was evaluated by measuring the amount of glucose released after treatment of tissue extracts with *Aspergillus niger* amyloglucosidase as described [53]. Tissues were fixed in 3% glutaraldehyde (EM grade, Electron Microscopy sciences, Hatfield, PA) in 0.2 M Sodium Cacodylate buffer for 4 h at 4°C, washed in 0.1 M Sodium Cacodylate buffer, and stored at 4°C in the same buffer. Samples were then embedded in paraffin, sectioned, and stained with periodic acid-Schiff (PAS) by standard methods.

### Force measurements

The overall muscle strength was evaluated by grip strength measurements using a grip strength meter (Columbus Instruments, Columbus, Ohio). The data were normalized by body weight and expressed as KGF/kg as described [54].

### Fiber Size Measurements

Tibialis anterior (fast) muscle was fixed in 4% paraformaldehyde, cross-sectioned, and stained with H & E (hematoxylin and eosin).

Bright field images of cross sections were obtained on a Leica DMR microscope (objective  = 10X/0.5 NA) with a QImaging Qcolor3 digital camera. Three to four representative frames per slide were analyzed for cross-sectional area of individual fibers. The quantifications were done with ImageJ (available at http://rsbweb.nih.gov/ij/). Muscle fiber size was measured in a comparable number of WT (n = 2261), KO (n = 3035), and tgKO (n = 2670) fibers. Two mice of each genotype were used for the experiments.

Student's *t* tests were used to compare LC3-II/LC3-I ratio, glycogen levels, and muscle strength.

All data are expressed as the mean ± SEM.

Animal care and experiments were conducted in accordance with the National Institutes of Health Guide for the Care and Use of Laboratory Animals.

## Supporting Information

Figure S1 **tgKO converted fibers do not show the Golgi complex distribution typical of slow fibers.**
Single muscle fibers from the psoas muscle of a tgKO mouse were stained with an antibody against GM130, a protein of the Golgi complex (green), and with the nuclear stain Hoechst 33342 (blue). Each panel represents a single confocal optical section from a different fiber, focused on the nuclei. The fiber in A shows Golgi elements which are dispersed or located at both poles of the nuclei (arrows), a pattern characteristic of type II (fast) organization [58]. The other panels show distributions that are characteristic of neither fast nor slow fibers, with occasional rare nuclei (arrowheads) surrounded by Golgi elements, a typical type I organization. Fibers D and E show dark channels which are the imprints of blood vessels, abundant around type I fibers. Bars: 10 µm.
(TIF)

Figure S2 **tgKO converted fibers have a microtubule distribution distinct from that of the fast or slow KO fibers.** Single muscle fibers were stained for microtubules (green) and lysosomes (red). Single confocal images are shown, focused on the core of the fibers. In both KO fibers, microtubules form long fascicles that surround the lysosomes and link them. This organization is found in occasional tgKO fibers (converted, 1) but most tgKO fibers (converted, 2) show short, disordered, microtubules. Bar: 10 µm.
(TIF)

Figure S3 **Single fibers (gastrocnemius; type II) from WT, KO, and tgKO stained for LC3 (the image is shown in black and white).** LC3-positive clusters of autophagosomes are found in the autophagic area of virtually every fiber in the KO. Pockets of LC3-positive autophagosomes can be found in some fibers in the tgKO. Bar: 10 µm.
(TIF)

Figure S4 **Leaky lysosomes in tgKO psoas fiber.** EM of psoas muscle, showing 2 lysosomes with spikes (arrowheads) showing leakiness. Bar: 500 nm.
(TIF)
