# Supplementary figures and images for: Fiber Type Conversion by PGC-1α Activates Lysosomal and Autophagosomal Biogenesis in Both Unaffected and Pompe Skeletal Muscle
Source: PLoS One. 2010 Dec 13;5(12):e15239. doi: 10.1371/journal.pone.0015239 (PMC3001465; doi:10.1371/journal.pone.0015239)

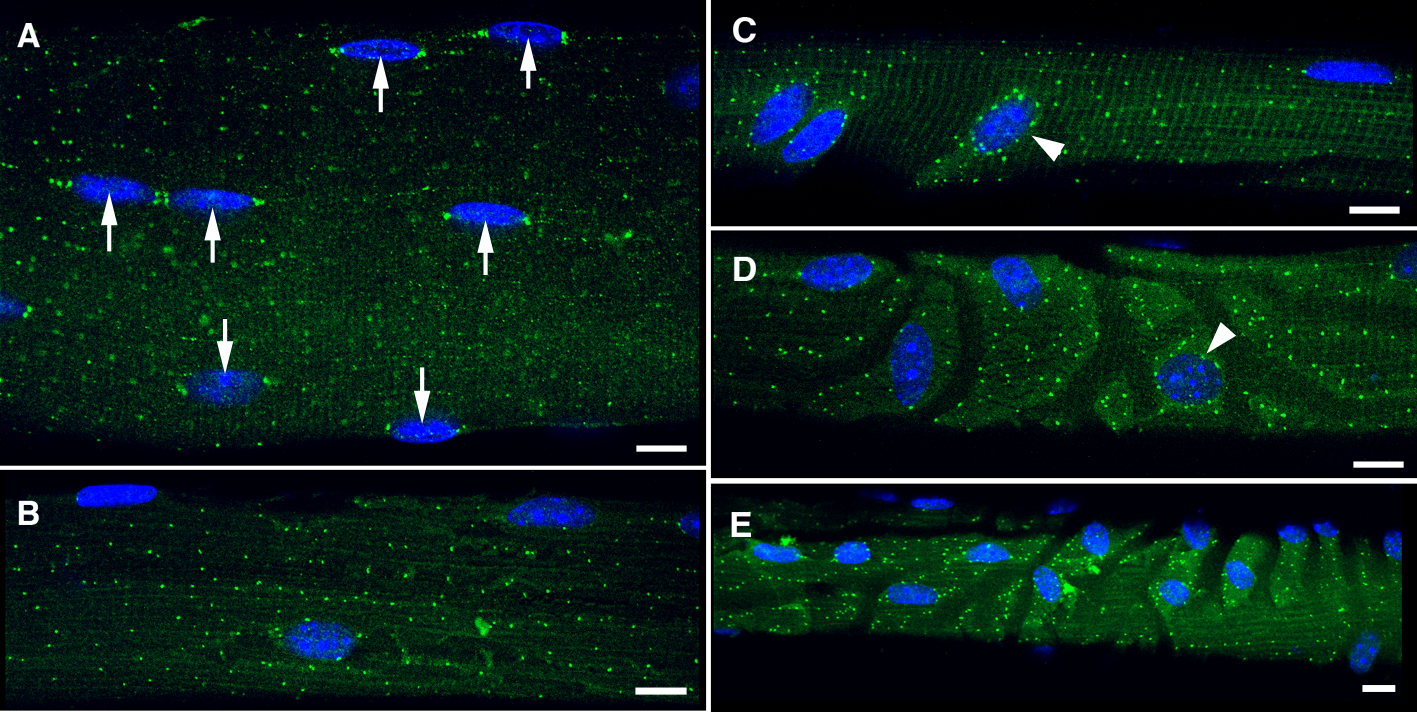

Supplement: Figure S1 — tgKO converted fibers do not show the Golgi complex distribution typical of slow fibers. Single muscle fibers from the psoas muscle of a tgKO mouse were stained with an antibody against GM130, a protein of the Golgi complex (green), and with the nuclear stain Hoechst 33342 (blue). Each panel represents a single confocal optical section from a different fiber, focused on the nuclei. The fiber in A shows Golgi elements which are dispersed or located at both poles of the nuclei (arrows), a pattern characteristic of type II (fast) organization [58]. The other panels show distributions that are characteristic of neither fast nor slow fibers, with occasional rare nuclei (arrowheads) surrounded by Golgi elements, a typical type I organization. Fibers D and E show dark channels which are the imprints of blood vessels, abundant around type I fibers. Bars: 10 µm. (TIF) [file pone.0015239.s001.tif]

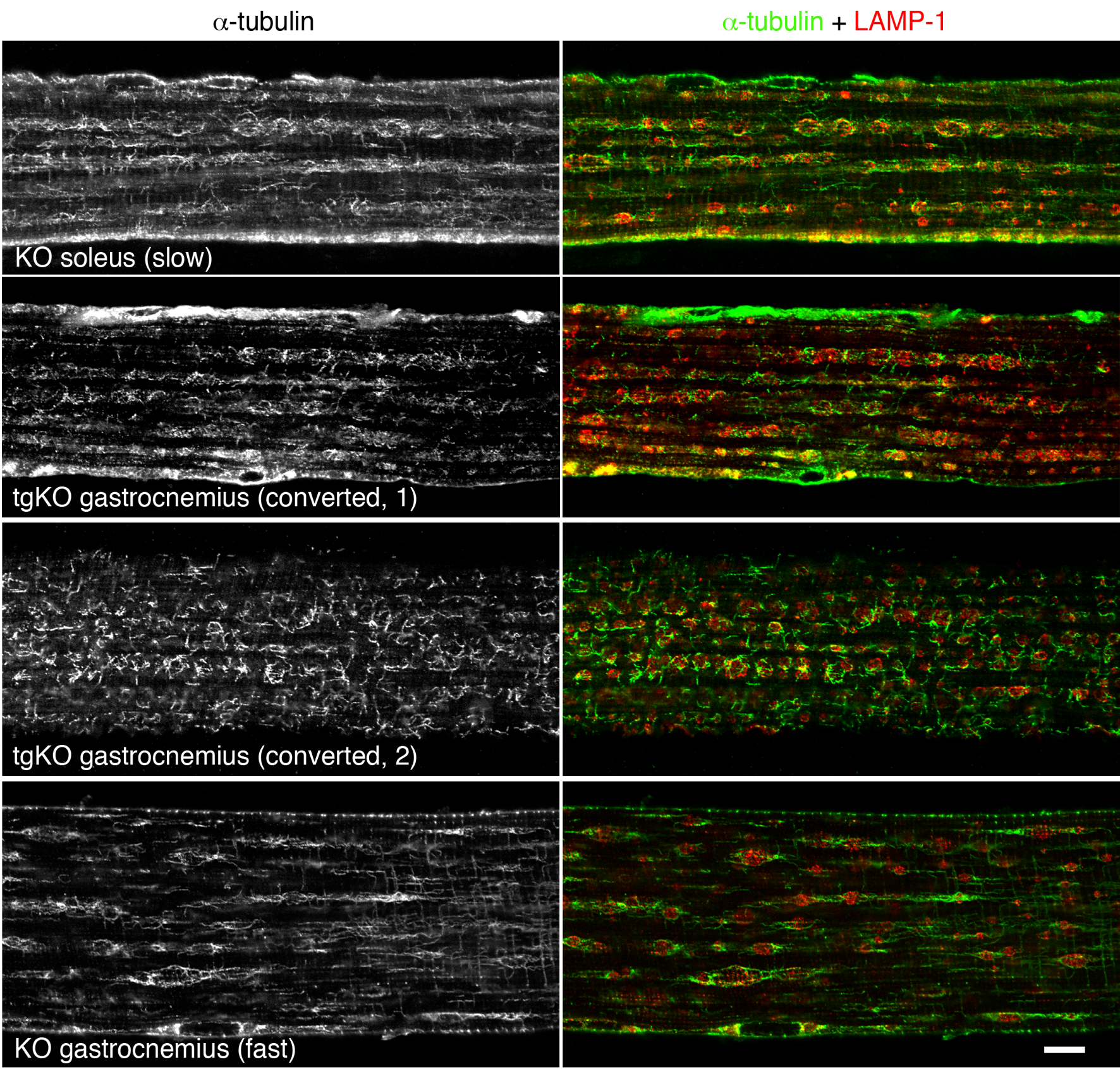

Supplement: Figure S2 — tgKO converted fibers have a microtubule distribution distinct from that of the fast or slow KO fibers. Single muscle fibers were stained for microtubules (green) and lysosomes (red). Single confocal images are shown, focused on the core of the fibers. In both KO fibers, microtubules form long fascicles that surround the lysosomes and link them. This organization is found in occasional tgKO fibers (converted, 1) but most tgKO fibers (converted, 2) show short, disordered, microtubules. Bar: 10 µm. (TIF) [file pone.0015239.s002.tif]

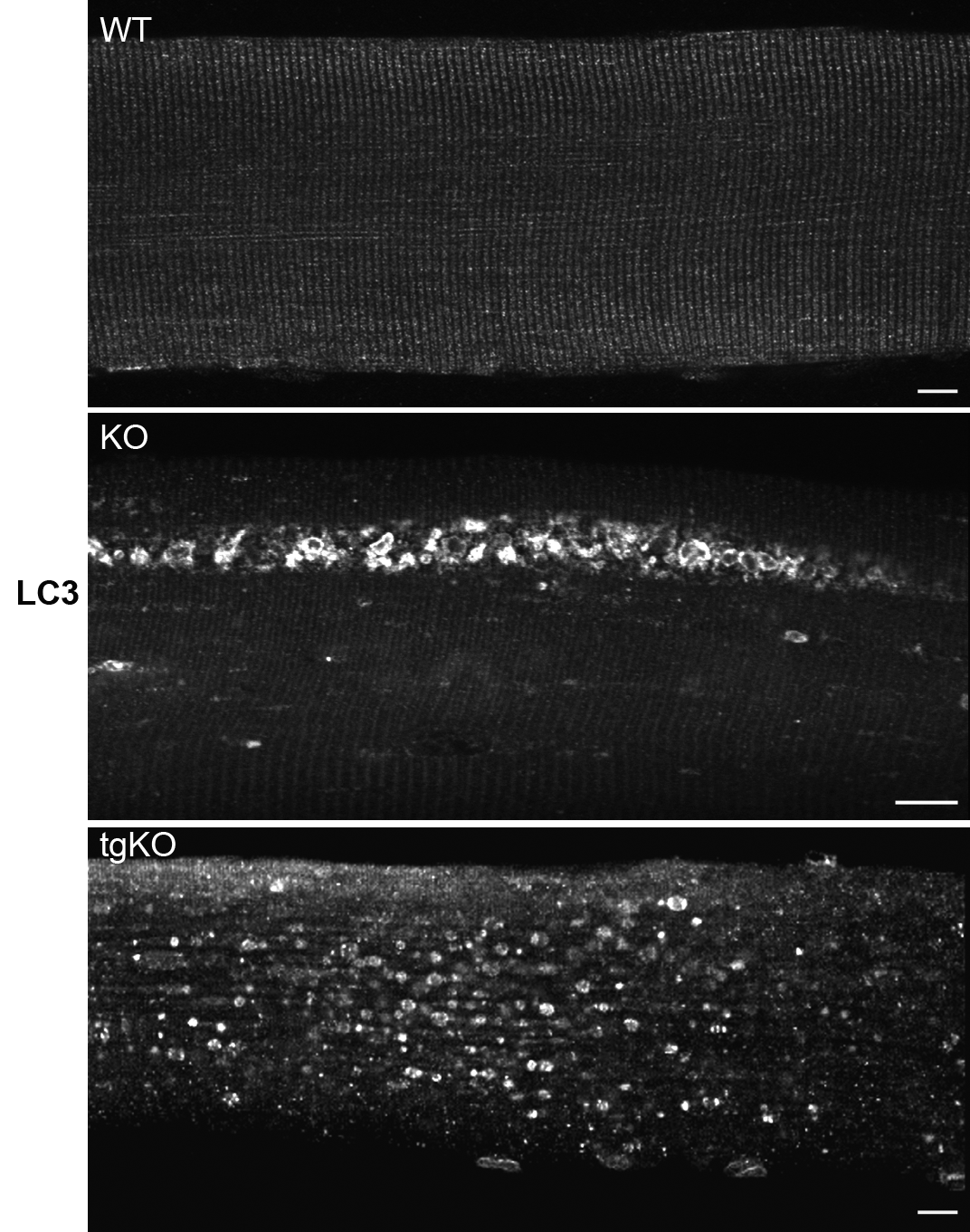

Supplement: Figure S3 — Single fibers (gastrocnemius; type II) from WT, KO, and tgKO stained for LC3 (the image is shown in black and white). LC3-positive clusters of autophagosomes are found in the autophagic area of virtually every fiber in the KO. Pockets of LC3-positive autophagosomes can be found in some fibers in the tgKO. Bar: 10 µm. (TIF) [file pone.0015239.s003.tif]

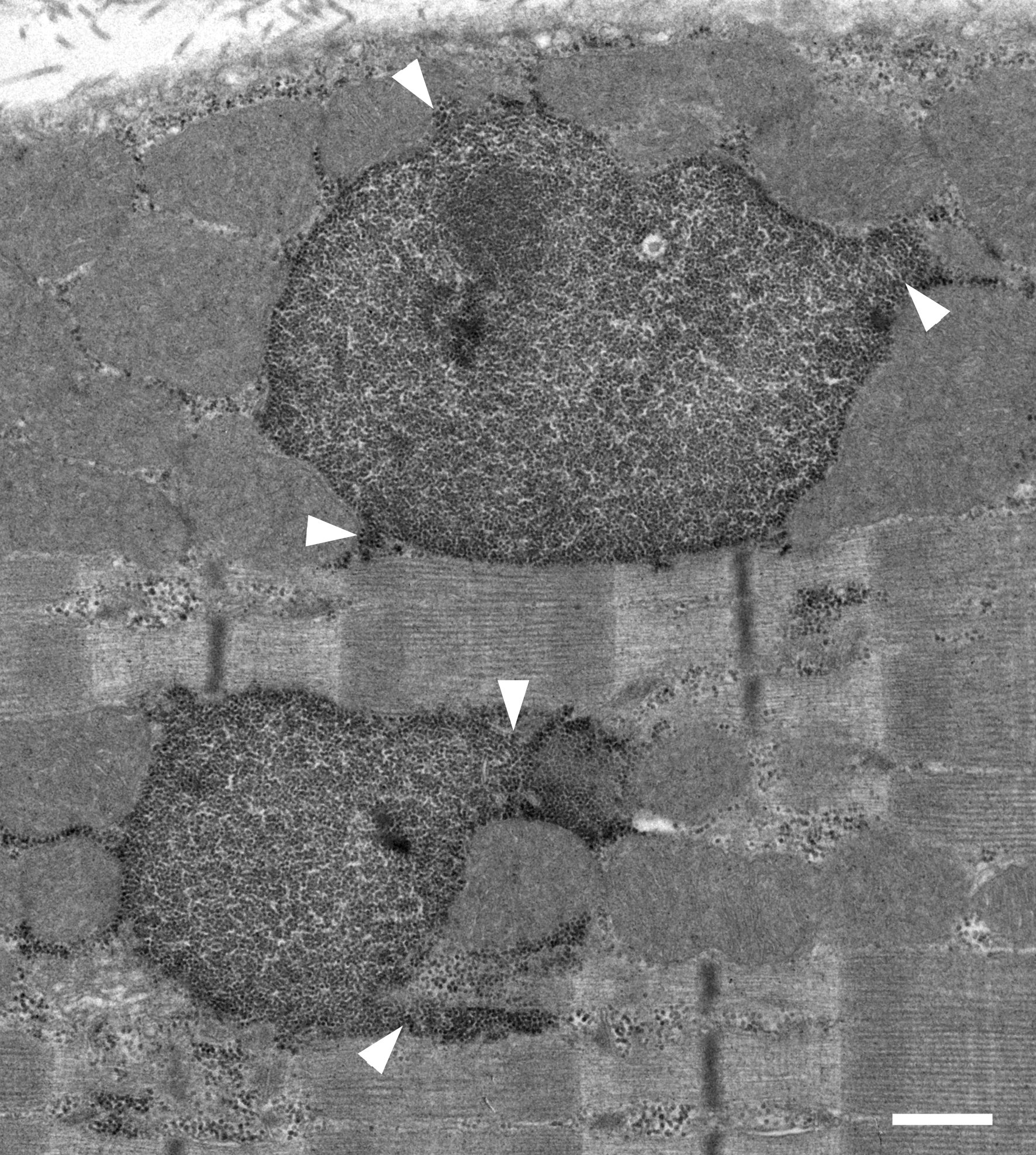

Supplement: Figure S4 — Leaky lysosomes in tgKO psoas fiber. EM of psoas muscle, showing 2 lysosomes with spikes (arrowheads) showing leakiness. Bar: 500 nm. (TIF) [file pone.0015239.s004.tif]
